# Placental Immune Tolerance and Organ Transplantation: Underlying Interconnections and Clinical Implications

**DOI:** 10.3389/fimmu.2021.705950

**Published:** 2021-08-03

**Authors:** Jin-Yu Sun, Rui Wu, Jiang Xu, Hui-Ying Xue, Xiao-Jie Lu, Jiansong Ji

**Affiliations:** ^1^Department of Cardiology, The First Affiliated Hospital of Nanjing Medical University, Nanjing, China; ^2^Department of Digestive Endoscopy, The First Affiliated Hospital of Nanjing Medical University, Nanjing, China; ^3^Department of General Surgery, The First Affiliated Hospital of Nanjing Medical University, Nanjing, China; ^4^Department of Rehabilitation, Huai’an Second People’s Hospital and The Affiliated Huai’an Hospital of Xuzhou Medical University, Huai’an, China; ^5^The Reproductive Center, Huai’an Maternal and Child Health Care Hospital, Xuzhou Medical University, Huai’an, China; ^6^Department of General Surgery, Liver Transplantation Center, The First Affiliated Hospital of Nanjing Medical University, Nanjing, China; ^7^Key Laboratory of Imaging Diagnosis and Minimally Invasive Intervention Research, Lishui Hospital of Zhejiang University/Fifth Affiliated Hospital of Wenzhou Medical University, Lishui, China

**Keywords:** placental immune tolerance, organ transplantation, immune rejection, clinical implication, maternal-fetal interface

## Abstract

The immune system recognizes and attacks non-self antigens, making up the cornerstone of immunity activity against infection. However, during organ transplantation, the immune system also attacks transplanted organs and leads to immune rejection and transplantation failure. Interestingly, although the embryo and placenta are semi-allografts, like transplanted organs, they can induce maternal tolerance and be free of a vigorous immune response. Also, embryo or placenta-related antibodies might adversely affect subsequent organ transplantation despite the immune tolerance during pregnancy. Therefore, the balance between the immune tolerance in maternal-fetal interface and normal infection defense provides a possible desensitization and tolerance strategy to improve transplantation outcomes. A few studies on mechanisms and clinical applications have been performed to explore the relationship between maternal-fetal immune tolerance and organ transplantation. However, up to now, the mechanisms underlying maternal-fetal immune tolerance remain vague. In this review, we provide an overview on the current understanding of immune tolerance mechanisms underlying the maternal-fetal interface, summarize the interconnection between immune tolerance and organ transplantation, and describe the adverse effect of pregnancy alloimmunization on organ transplantation.

## Introduction

The immune system recognizes, targets, and attacks non-self antigens, which makes up the fundamental of immunity activity against infection (1). However, the immune system also attacks transplanted organs, leading to immune rejection and transplant failure (2). The developing embryo and placenta could be considered semi-allografts (3), while it can induce maternal tolerance and be free of a vigorous immune response (4). The balance between the immune tolerance in the maternal-fetal interface and normal infection defense plays a fundamental role in pregnancy (5), which inspires further research in the immune tolerance of maternal-fetal interface. Accumulating studies suggest that the tolerogenic mechanisms in the maternal-fetal interface are associated with and might be applied in organ transplantations. This review provides an overview of the current understanding of immune tolerance mechanisms underlying the maternal-fetal interface. Then, we summarize the interconnection between immune tolerance and organ transplantation. Finally, we describe the adverse effect of pregnancy alloimmunization on organ transplantation and discuss the current challenges.

## Immune Tolerance Mechanisms Underlying the Maternal-Fetal Interface

During pregnancy, the non-functional state of the primary immune cells (including decidual natural killer [dNK] cells, decidual T cells, and decidual macrophages) induces placental immune tolerance, and the dNK cells are probably the most important in the many immune cells (6). Besides, immune tolerance synergistic molecules are another significant players in the maternal-fetal interface, such as HLA molecule and co-signaling molecules. HLA molecule is usually involved with host *versus* graft reaction, while the non-classic major histocompatibility complex (MHC) class I molecule, HLA-G, was revealed to induce immune tolerance in the maternal-fetal interface. Co-signaling molecules enhance the biological function of trophoblasts, decidual stromal cells, and decidual immune cells, while recent studies also revealed their roles in regulating placental immune tolerance.

### The Role of dNK Cells in Placental Immune Tolerance

CD56^bright^CD16^−^ NK cells are prominent NK cells (about 70%-80% of lymphocytes), which make up more than half of the maternal immune cells in human early pregnancy decidua (6). The origin of NK cells in the endometrium remains unclear. The C-X-C chemokine ligand 12 (CXCL12)/C-X-C chemokine receptor type 4 (CXCR4) axis is necessary to recruit dNK cells at the maternal-fetal interface. Tao et al. (7) obtained the decidual tissues from normal pregnancies (5-10 weeks) and revealed that CXCL12/CXCR4 axis could facilitate the migration of dNK cells concentration-dependent manner. CXCL12 could increase the migration of dNK cells by 1.2-, 1.4-, and 1.9-fold at a concentration of 1, 10, and 100 ng/ml, respectively (7). When trophoblast culture media were treated with anti-CXCR4, the chemotactic activity of dNK cells was significantly inhibited (*P* < 0.01) (7).

**Figure 1:**
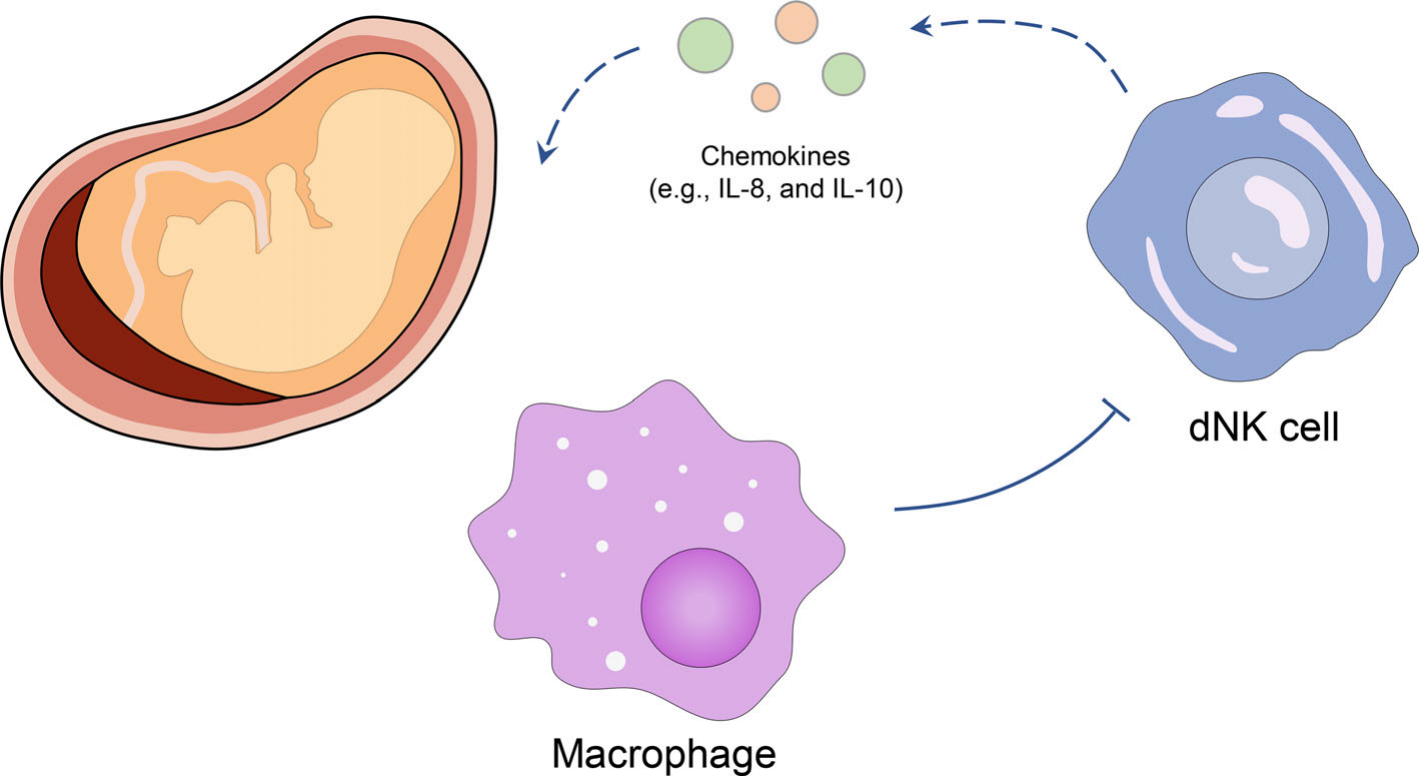
Inhibitory effect of macrophages on dNK cells in the maternal-fetal interface. Macrophages induce the immune tolerance of dNK cells and decrease the chemokines (such as IL-8, and IL-10) in the maternal-fetal interface, thus stabilizing the embryo.

DNK cells were reported to play a central regulating role in decidual tolerance to embryos. During the first trimester, dNK cells participate in trophoblast invasion by producing chemokines (such as IL-8 and IL-10) without attacking placental cells (8, 9) since they would not polarize granules to the target cells (10). Co et al. (11) first revealed that the inhibited killing of dNK cells in invasive cytotrophoblasts was induced by maternal decidual macrophages cell, which provided a possible reason for the anergic dNK cells in placental immune. Placental (5-22 weeks) and decidual tissues (5-13 weeks) were achieved from patients undergoing elective terminations of pregnancy, and the dNK cells were cultured *in vitro*. The results showed that dNK cells could kill K562 target cells (leukemia cell lines) after removing macrophages, while the cytotoxicity of dNK cells was inhibited again when macrophages were added back. Furthermore, the addition of soluble TGF-β1 to cytotoxicity assays had a significant inhibitory effect on purified dNK-induced cytotoxicity at a lower dNK: K562 ratio (5:1 *vs.* 20:1), and more primary cytotrophoblast targets (such as NK92 and K562) were attacked after removal of TGF-β1, which suggested that the immune tolerance of dNK cells could be induced by TGF-β1 (12). Wang et al. (13) obtained villi from healthy women receiving pregnancy termination (7-9 weeks) and extracted primary culture of first-trimester trophoblast cells. They reported that CXCL16 induced the polarization of M2 macrophages *via* inducing high IL-10 expression. Moreover, when dNK cells were co-cultured with M2 macrophage pretreated with rhCXCL16, the decreased expression of IL-15 facilitated the inactivation of dNK cells. These studies indicate the promoting role of macrophages in the dNK cell-induced immune tolerance at the maternal-fetal interface (**Figure 1**).

Recently, Huang et al. (14) recruited recurrent spontaneous abortion women (n=49) and normal pregnant women (n=52) and measured the expression of miR-30e in the decidua tissues *via* a quantitative polymerase chain reaction. They revealed that the expressions of miR-30e in individuals with recurrent spontaneous abortion were significantly decreased compared with the control group, and the cytotoxicity of dNK cells was reduced by upregulating miR-30e. Additionally, Tirado-Gonzalez et al. (15) reported that dNK cells induced apoptosis in decidua dendritic cells during pregnancy (40.2 ± 7.2% of DC-SIGN^+^ cells in decidual sections and 34.4 ± 15.2% in leukocyte suspensions), which induced the immune tolerance environment for the maternal-fetal interface.

Besides maintaining fetal tolerance, dNK cells might also contribute to the placental infection defense (16). Crespo et al. collected placental and decidual samples (6-12 weeks) and revealed that the antimicrobial peptide granulysin was highly expressed in human dNK cells. Antimicrobial peptide granulysin was selectively transferred *via* nanotubes to extravillous trophoblasts (EVTs) to kill intracellular Listeria monocytogenes without killing the trophoblasts (17, 18). Also, when pregnant women were infected with human cytomegalovirus (HCMV), the expression of killer cell immunoglobulin-like receptor 2DS1 (KIR2DS1) by dNK cells increased the ability to prevent placental HCMV infection. Similarly, the interaction between KIR2DS1 and HLA-C2 could enhance the activation of KIR2DS1 single-positive dNK and improve the response to placental HCMV infection (6-12 weeks human placental and decidual material) (19).

### The Role of Human Leukocyte Antigen in Placental Immune Tolerance

**Figure 2:**
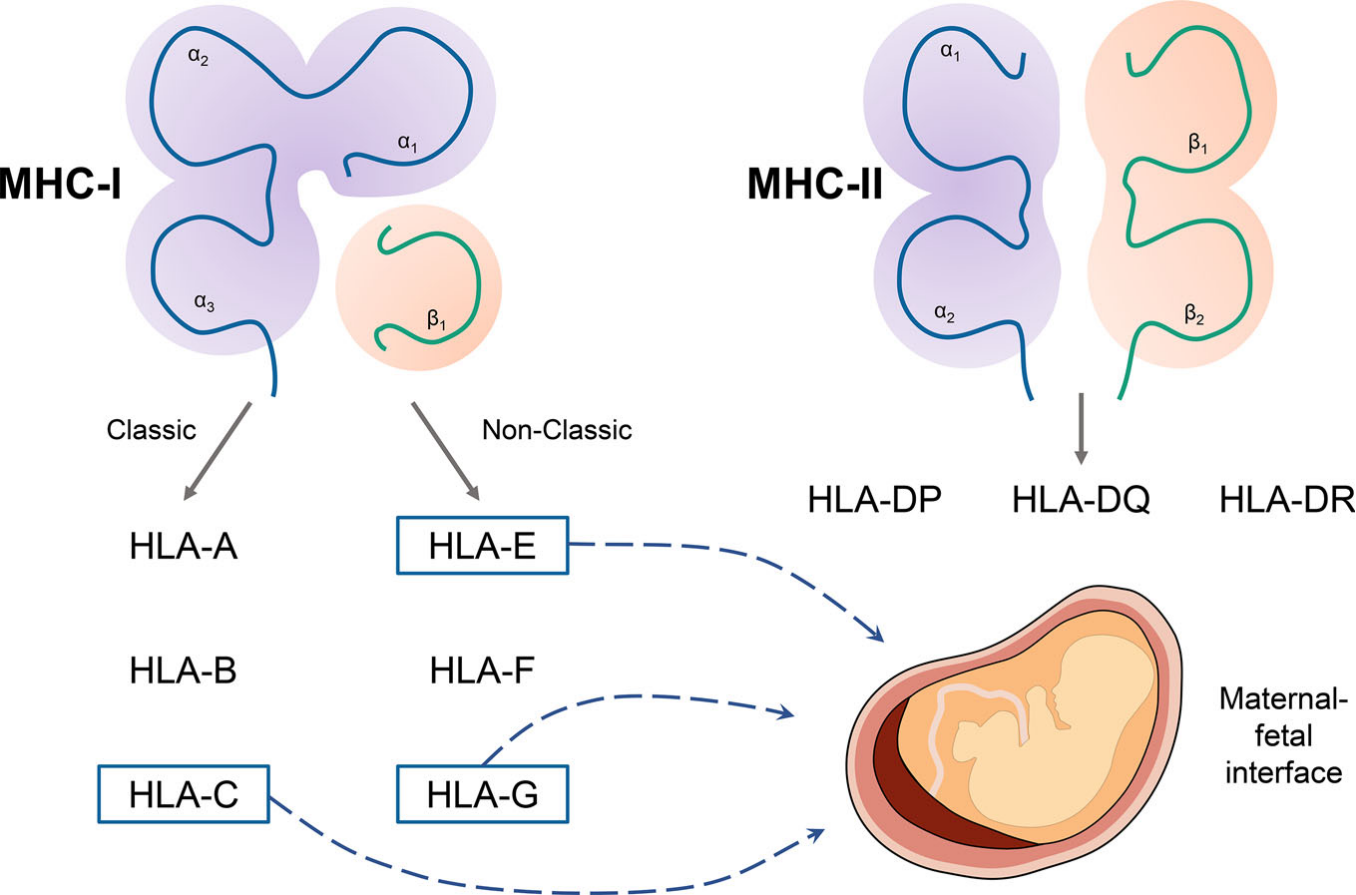
The classification of major histocompatibility complex (MHC) molecules and main molecules in maternal-fetal immunity. MHC includes MHC class I and II molecules. Classic MHC class I molecules include human leukocyte antigen (HLA)-A, HLA-B, and HLA-C. The non-classic MHC class I molecules include HLA-E, HLA-F, and HLA-G. MHC class II molecules include HLA-DR, HLA -DP, HLA -DQ. HLA-C, HLA-E, and HLA-G play a significant role in immune tolerance in the maternal-fetal interface.

Recently, accumulating studies are focused on the role of HLA in maternal-fetal immunity, especially HLA-G. EVT expresses a unique type of MHC molecules to induce immune tolerance. The tripartite interactions of human leukocyte antigen (HLA)-peptide-T cell receptors are fundamental in enabling the adaptive immune system, and MHC class I and II molecules could distinguish the self from the non‐self antigen (**Figure 2**).

Regulation of HLA expression patterns is another potential strategy to inhibit organ transplantation rejection (20). Unlike most cells, EVTs do not express ubiquitous classical MHC class I molecules (i.e., HLA-A and HLA-B) (21). Instead, HLA-C and the nonclassical MHC class I molecules (i.e., HLA-E and HLA-G) are expressed in EVTs. HLA‐G shows great immunosuppressive features and a limited expression outside the trophoblasts (1). In the term pregnancy, HLA-G^+^ EVTs have a weakened capacity to induce Treg *via* isolating three types of HLA-G^+^ EVTs from human placental tissues (HLA-G^+^ EVTs, 6 to 12 weeks, n = 2; HLA-G^+^ EVTs, >37 weeks, n = 3; HLA-G^+^ chorionic EVTs, >37 weeks, n = 3), suggesting the distinct ability of HLA-G to enhance placental immune tolerance *via* directly inducing Treg cells (22). Meanwhile, the HLA-G could interact with decidual T cells, NK cells, macrophages, and dendritic cells and induce immune tolerance (23–25). Interestingly, Bortolotti et al. (26) isolated mesenchymal stromal/stem cells (MSCs) in endometrial decidual tissue (EDT) from menstrual blood collected from 5 healthy women during the first few days of the menstrual cycle. They reported that EDT-MSC could inhibit lymphocytes proliferation (70% for low culture passage EDT-MSC, 40% for high culture passages EDT-MSC, *P* < 0.002). Interestingly, when human EDT-MSCs were infected by the human herpesvirus (HSV)-1, the expression of HLA-G and its immunosuppressive function will be significantly reduced in a dose-dependent manner.

Meanwhile, HLA-G is closely related to dNK cells in the maternal-fetal interface. Tilburgs et al. (27) collected human placental and decidual material (6-12 weeks), and the results showed that co-culture of activated dNK with EVTs could lead to the reacquisition of surface HLA-G. Meanwhile, co-culture of HLA-G-expressing melanoma M8 cells with dNK cells could restore the killer cell immunoglobulin-like receptor 2DL4 or immunoglobulin-like transcript 2 expression on dNK cells, which increased the expression levels of surface HLA-G. Therefore, the activation of dNK could decrease the internalized HLA-G and restore the cytotoxicity, and the HLA-G could induce tolerance of dNK in turn.

### The Role of Co-Signaling Molecules in Placental Immune Tolerance

Generally, immune cells can be fully activated after a second signal (co-signal) derived from the interaction between receptors on immune cells and the corresponding co-stimulating ligands on antigen-presenting cells. This classic ‘two-signal model’ is originated from the model of lymphocyte activation, which functions to optimize the immune response (28, 29). The potential co-signaling molecules mediating maternal-fetal immune tolerance are summarized in **Table 1**, including programmed death 1 (PD-1)/programmed death-ligand 1 (PD-L1) (30–34), T cell immunoglobulin and mucin domain 3 (TIM-3) (35–40), cytotoxic T-lymphocyte-associated antigen 4 (CTLA-4) (36, 37, 41), inducible co-stimulator (ICOS)/inducible co-stimulator ligand (ICOS-L) (42), carcinoembryonic Ag cell adhesion molecule 1 (CEACAM1) (43), and leukocyte-associated immunoglobulin like receptor-1 (LAIR-1) (44).

**Table 1:** Co-signaling molecules and their mechanism to induce the maternal-fetal immune tolerance.

Cells	Co-signaling Molecules
**Trophoblasts**	Syncytiotrophoblasts: PD-L1, CD200, CD200R1, PD-L2
	Cytotrophoblasts: PD-L1, CD200, CD200R1
	EVTs: ICOS-L, PD-L1, CD276, CEACAM1, Gal-9, CD200, CD200R1, CD155, CD112
**Decidual stromal cells**	TIM-3, Gal-9, PD-L1, PD-L2
**Decidual immune cells**	dNK cells: CEACAM1, TIM-3, LAIR-1, CD226
	Decidual T cells: PD-1, CEACAM-1, TIM-3, LAIR-1
	Decidual macrophages: LAIR-1
**Main Molecules**	**Recent studies**
**PD-1/PD-L1**	Elevated PD-1 expression in decidual CD8^+^ T, CD4^+^ T, and NKT-like cells and PD-L1 expression in decidual CD4^+^ T, Treg, NKT-like and CD56^+^ NK cell compared to peripheral blood (30).
	The soluble PD-L1 increased in pregnant women and suppress maternal immunity (31).
	Blockade of PD-1 resulted in decreased proliferation and Th2-type cytokine production while increased trophoblast killing and IFN-γ producing capacities of CD8^+^ T cells (32).
	PD-1/PD-L1 signaling is critical for macrophage differentiation and function, which is the success of a pregnancy (33).
	PD-1 promote Th2 bias and pregnancy maintenance by regulating CD4^+^ T cell function at the maternal-fetal interface (34).
**TIM-3**	Decidual NKT cells exhibit a reduced TIM-3 expression with increased relative receptor expression and a slightly increased cytotoxicity when compared to the periphery (35).
	TIM-3^+^CTLA-4^+^dCD8^+^ T cells produced more anti-inflammatory cytokines. Blocking TIM-3 pathways inhibited the anti-inflammatory cytokines and induced fetal loss (36).
	TIM-3 pathways maintain tolerance by regulating dCD4^+^T cells. Blockade of TIM-3 pathways induces fetal loss with altered cytokine profiles by dCD4^+^ T cells (37).
	TIM-3 is upregulated in NK cells and inhibits NK cytotoxicity toward trophoblast in Gal-9 dependent pathway (38).
	Activation of TLR signaling induced upregulated TIM-3 expression. TIM-3 inhibited TLR signaling-induced inflammatory cytokine production (39).
	TIM-3 are expressed on over 60% of dNK cells. TIM-3^+^ dNK cells display higher IL-4 and lower TNF-α and perforin production.
	Peripheral NK cells can be transformed into a dNK-like phenotype *via* Gal-9 and the interaction between Gal-9 and TIM-3.
	Trophoblasts inhibit LPS-induced pro-inflammatory cytokine and perforin production by dNK cells, which can be attenuated by TIM-3 neutralizing antibodies.
	Th2-type cytokines decreased and Th1-type cytokines increased in TIM-3^+^ dNK cells from human and mouse miscarriages (40).
**CTLA-4**	Blocking CTLA-4 pathways inhibited the anti-inflammatory cytokines and induced fetal loss (36).
	CTLA-4 pathways maintain tolerance by regulating dCD4^+^T cells. Blockade of CTLA-4 pathways induces fetal loss with altered cytokine profiles by dCD4^+^ T cells (37).
	Antigen-stimulated T cells become activated ligated with CD28 and anergic ligated with CTLA-4 (41).
**ICOS/ICOS-L**	ICOS-L blockade abrogates placental immune tolerance by enhancing CD8^+^ effector response and reducing local immunomodulation *via* CD8^+^ Treg cells (42).
**CEACAM1**	CEACAM1 interactions inhibit the lysis, proliferation, and cytokine secretion of activated dNK, T, and NKT cells, respectively (43).
**LAIR-1**	Co-culture of dNK with primary TROs/DSCs downregulated Th1 cytokine production, which were abrogated by LAIR-1 inhibitor (44).

PD-L1, programmed death-ligand 1; CD200, cluster of differentiation-200; CD200R1, CD200 receptor 1; PD-L2, programmed death-ligand 2; ICOS-L, inducible co-stimulator ligand; CD276, cluster of differentiation-276; CEACAM1, carcinoembryonic Ag cell adhesion molecule 1; Gal-9, galectin-9; CD155, cluster of differentiation-155; CD112, cluster of differentiation-112; TIM-3, T cell immunoglobulin and mucin domain 3; LAIR-1, leukocyte-associated immunoglobulin-like receptor-1; CD226, cluster of differentiation-226.

To the best of our acknowledge, PD-1/PD-L1 is probably the most studied among the many co-signaling molecules in maternal-fetal immunity (45). Meggyes et al. (35) used flow cytometry to measure PD-1 expression by peripheral and decidual immune cells from pregnant BALB-c mice on day 14.5 of pregnancy. The expression of PD-1 significantly increased in dNK cells (*P* = 0.001), γ/δ T cells (*P* = 0.002), and NKT cells (*P* = 0.024), thus reducing cytotoxic potential compared with the periphery. Interestingly, the upregulation of PD-1 was consistent with the increase in maternal T cells in the decidua, which indicated a potential role for the PD-1/PD-L1 axis in silencing maternal immune responses to fetal antigens (46). In the following study by Costa et al. (47), the term (37-40 weeks), uncomplicated gestation placentas were obtained, and the results revealed that the PD-1/PD-L1 axis might limit T cell expansion and induce tolerance to the fetal allotransplant. Wang et al. (32) obtained the villous and decidual tissues from normal (n = 78) and miscarriages (n = 36) pregnancy. They reported that the co-culture of CD8^+^ T cells and trophoblasts could upregulate PD-1^+^ immune cells (*P* < 0.001). Blockade of Tim-3 and PD-1 decreased CD8^+^ T cell proliferation, enhanced trophoblast killing, and increased IFN-γ production by CD8^+^ T cells. Then, they established a mouse pregnancy model by mating BALB/c males with CBA/J females and reached the same conclusion *in vivo*. Sayama et al. (48) reported that production of IFN-γ was suppressed in peripheral T cells when T cell-expressed PD-1 and macrophage-expressed PD-L1 interacted (Human first-trimester decidual samples, n = 10), while the suppressive property was not observed in the monocytes lacking PD-L1. These studies demonstrated that PD-1/PD-L1 mediated immune tolerance as the co-signaling molecules. Besides, PD-L1 was reported to directly foster Treg differentiation and induce immune tolerance (49).

## The Relationship Between Placental Immune Tolerance and Organ Transplantation

Since HLA-G can maintain placental immune tolerance during pregnancy, it has been well explored as the promising diagnostic and even therapeutic target of organ transplantation. Ajith et al. (50) compared the expression of soluble HLA (sHLA)-G in patients with the immune rejection of the renal allograft (n=40) and those with a functioning renal allograft (n=90). The results showed that HLA-G could inhibit the activation and cytotoxic capabilities of human CD8^+^ T cells, one of the important mechanisms in organ transplantation rejection. Furthermore, Janssen et al. (51) genotyped 41 kidney recipients with acute rejection and 134 without rejection, and the results suggested that HLA-G polymorphisms were higher in recipients without acute rejection. Similarly, Durmanova et al. (52) reported that HLA-G polymorphisms were closely associated with graft acceptance. The sHLA-G level can predict the renal allograft outcome for high expression in patients with renal allograft (31.6 ± 20.2 *vs.* 17.7 ± 8.5, *P*=0.003). These studies suggest that HLA-G and its polymorphisms might play a potential protective role in organ transplantation (53). However, another study did not support the protective effect of HLA-G in liver transplantation since sHLA-G levels were higher in recipients with acute rejection than nonrejectors (54). The authors claimed that it might be negative feedback to protect the liver against immunological damage (54).

Apart from the acute rejection, HLA-G can also predict the long-term prognosis of organ transplantation. Lazarte et al. (55) reported that sHLA-G increased in patients with chronic lung allograft dysfunction, and HLA-G single nucleotide polymorphism +3142 was associated with increased mortality (hazard ratio [HR] = 1.78, *P* = 0.015). Consistently, Brugiere et al. (56) and Ezeakile et al. *(* 57) demonstrated that the HLA-G level and expression of the membrane-bound form of HLA-G on monocytes were related to the postoperative lifetime of kidney transplantation. Additionally, Adamson et al. (58) also found that HLA-G was a risk factor for cell-mediated rejection (CMR) following heart transplant in humans with a *P*-value of 0.03 (n = 123). Compared with the minor HLA-G +3196/GG genotype, the CG genotype had a 47.2% reduction in CMR risk (HR = 0.528, 95% CI, 0.235-1.184), while CC genotype had a 66.9% reduction (HR = 0.331, 95% CI, 0.144-0.761). The authors indicated that HLA-G might be considered a diagnostic strategy and a potential therapeutic target for transplant rejection. In the following research, Von et al. (59) firstly reported the therapeutic recombinant HLA-G5 in the intestinal transplantation model. Allogenic intestinal transplantation was performed in rats (Brown Norway to Lewis) with and without HLA-G treatment. The rats with HLA-G treatment showed significantly decreased postoperative acute rejection in 4 and 7 days after the operation, and acute rejection-related gene expression was higher in rats with HLA-G treatment (TNFα, *P* < 0.05; IL-10, *P* < 0.05).

Moreover, NK cells and co-signaling molecules also induce immune tolerance in organ transplantation. For example, NK cells can induce both immune rejection and tolerance in liver transplantation (60). The recipient-derived NK cells tend to promote immune rejection, while the donor-derived NK cells tend to induce immune tolerance. Yazdani et al. (61) reported that the differential expression genes, which were identified in antibody-mediated rejection through bioinformatics pipeline (microarray transcriptomic data from a case-control study, n=95), were enriched in NK cell pathways. Also, in multivariate cox analysis, NK cell infiltration could predict graft failure (*P* < 0.001) and diagnosis of rejection (*P* = 0.039) with the highest accuracy compared with other immune cell subtypes (e.g., CD8^+^ T cells, CD4^+^ T cells, macrophages, and so forth) according to the Banff classification.

Furthermore, the PD-1/PD-L1 axis induces placental immune tolerance and improves organ transplantation. In brief, the PD‐1/PD‐L1 axis can induce tolerance in organ transplantation through PD‐1 and CD28 gates and influence the effector T cells. Xu et al. (62) reported that cellular exosome-like nanovesicles could inhibit the proliferation of mononuclear cells in peripheral blood (76% *vs*. 2%, *P* < 0.001) through the interaction of PD-1/PD-L1 and CTLA-4/CD80, which would subsequently decrease the density and activation of CD8^+^ T cells, downregulate cytokine production (HEK293T cells), and prolonged the survival of mouse skin and heart grafts. The blocked PD-1/PD-L1 axis could lead to a high rejection rate [37% to 80% (63)] for transplanted organs. A recent meta-analysis (64) reviewed the VigiBase database to explore the association of rejection events with drugs and revealed that anti-PD-1 and anti-PD-L1 drugs were more involved compared with anti-CTLA-4 drugs in rejection events (93.0% *vs*. 7.0%) due to the blockade of the specific pathway.

## The Adverse Effect of Pregnancy Alloimmunization on Organ Transplantation

Despite the immune tolerance during pregnancy, alloimmunization also exists, which might induce an increased risk for future organ transplantation rejection (65). Studies (66–71) regarding the adverse effect of pregnancy on subsequent organ transplantation are summarized in **Table 2**.

**Table 2:** Adverse effect of pregnancy on subsequent organ transplantation.

Author	Type	Study time	Sample size	Type of organ	Conclusion
Cohen et al. (66)	Retrospective	2001‐2013	5012	Kidney	No difference in graft failure between recipients of fathers and mothers.
Bromberger et al. (67)	Retrospective	2007‐2013	502	Kidney	Pregnancy is the major reason for loss of living donor access for women.
Redfield et al. (68)	Dataset analysis	1997‐2014	107292	Kidney	Pregnancy alone is made up 20% of sensitization; Waiting time for organ transplantation is longer for women.
Higgins et al. (69)	Retrospective	2003‐2012	64	No mention	Pregnancy leads to the greatest increase in HLA antibody levels from pre-treatment to peak
Choi et al. (70)	Retrospective	1979‐2011	374	Kidney	The antibody originated from the sensitization in pregnancy results in the transplantation failure.
Ghafari et al. (71)	Retrospective	1989‐2006	171	Kidney	Graft survival time was significantly worse because of pregnancy

HLA, human leukocyte antigen.

A study including 69 participants based on the Luminex screening test showed that about 24% to 49% of parous transplant candidates have the detectable anti‐HLA antibody, which composed a barrier to transplant success due to pregnancy alloimmunization (72). Redfield et al. (68) reported that retransplants (53%), pregnancy (20%), and transfusion (5%) were the three main reasons for high sensitivity to kidney transplantation (patients with a panel reactive antibody ≥ 98%). The rate of organ transplantation rejection increased in highly sensitized patients within one year compared with those non-sensitized (10.6% *vs*. 8.3%, *P* < 0.001). The 10-year survival rate of highly sensitized patients was also significantly worse (43.9% *vs*. 52.4%, *P* < 0.001). A study of 2,587 kidney transplant candidates suggested that the rate of living donor kidney transplantation for women decreased by 30% due to histocompatibility (67). Retransplants, pregnancy, and transfusion all contribute to the high sensitivity to transplantation, and retransplants have a more significant immunologic impact, followed by pregnancy and transfusion (73). However, in practice, spouses are often the primary transplant source, and pregnancy is the unique cause of sex disparity in organ transplantation (67).

Meanwhile, Van et al. (74) reported that the incidence of kidney graft loss was 9.4% within two years, 9.2% within 2-5 years, and 22.3% within 5-10 years after pregnancy, respectively. They pointed that pregnancy affected the graft loss significantly within two years. Furthermore, when comparing male and female recipients with 0% panel reactive antibody, there was no significant difference in all‐cause graft failure (66), which indirectly indicated that pregnancy alloimmunization might negatively impact the subsequent transplantation process after pregnancy.

## Conclusion

Currently, the survival of patients receiving organ transplantation remains unsatisfactory, with a five-year survival of 89.6% (75), 86.6% (76), and 77% (77) in the liver, kidney, and heart transplantation, respectively. Although immunosuppression has been widely used to inhibit acute graft-*vs*-host disease after organ transplantation (78), many complications (like heart failure or acute kidney injury) increase morbidity and mortality (79). Long-term immunosuppression treatment is likely to cause many graft- or host-related adverse events, such as bone mass loss (80), infection (81), and malignancies (82). Patients with organ transplants have an approximately 2-fold increased cancer risk than the general population (83, 84). The interconnection between placental immunity and transplantation immunity has recently become a hot topic since it might provide a potential strategy to regulate the immune balance between the hosts and transplanted organs. This article provides an overview on the current understanding of immune tolerance mechanisms underlying the maternal-fetal interface. We review the immune tolerance mechanisms of dNK cells and immune tolerance synergistic molecules (such as HLA molecule and co-signaling molecules), and we summarize current evidence on the relationship between immune tolerance and organ transplantation. Finally, we describe the adverse effect of pregnancy alloimmunization on organ transplantation.

**Figure 3:**
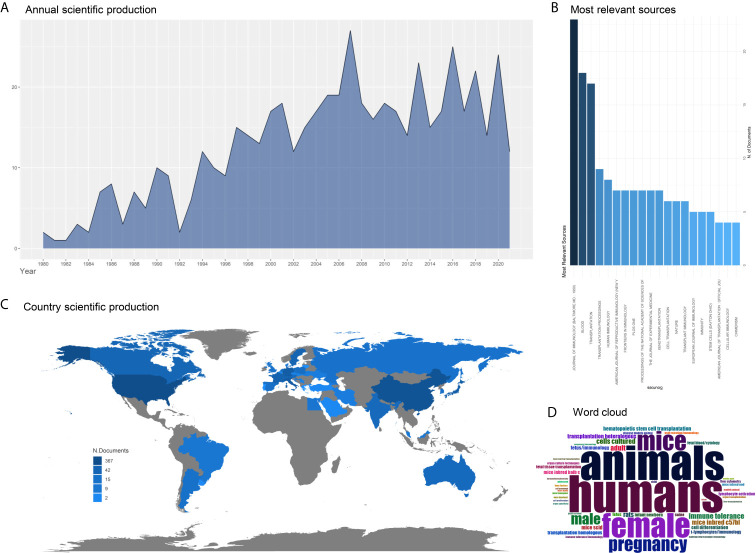
Trends of the research in placental immunity and organ transplantation. The search strategy is ((((((fetal) OR (placenta*)) OR (maternal-fetal interface)) AND (immune)) AND (organ)) AND (transplantation)) AND ((“1980”[Date - Publication]: “2021”[Date - Publication])). **(A)** The annual scientific production shows an increasing trend from 1980 to 2021. **(B)** The most relevant sources of these published studies. The top three relevant sources are *Journal of immunology*, *Blood*, and *Transplantation*. **(C)** Country scientific production. The color indicates the number of the related studies in each country. **(D)** Word cloud is based on the keywords from the published studies.

Still, it should be highlighted that the total view of the mechanisms underlying placental immunity remains vague. The immune tolerance in the maternal-fetal interface is a complex balance associated with both the immune tolerance molecules and immune microenvironment. However, most studies were performed on a cellular level, and there lacks enough validation in mature animal models. Only a few immune tolerance molecules in the maternal-fetal interface have been studied. Besides, the studies regarding the application of placental immunity on organ transplantation are limited (**Figure 3**). Accordingly, further *in vivo* experiments using animal models are necessary, and more immune pathways and immune networks should be explored.

## Author Contributions

J-YS, RW, and X-JL developed the study concept. RW wrote the manuscript with the help of J-YS, JX, H-YX, X-JL, and JJ. All authors contributed to the article and approved the submitted version.

## Conflict of Interest

The authors declare that the research was conducted in the absence of any commercial or financial relationships that could be construed as a potential conflict of interest.

## Publisher’s Note

All claims expressed in this article are solely those of the authors and do not necessarily represent those of their affiliated organizations, or those of the publisher, the editors and the reviewers. Any product that may be evaluated in this article, or claim that may be made by its manufacturer, is not guaranteed or endorsed by the publisher.
